# L1CAM High Expression Associates with Poor Prognosis in Glioma but Does Not Correlate with C11orf95-RELA Fusion

**DOI:** 10.1155/2020/1353284

**Published:** 2020-05-16

**Authors:** Jing Zeng, Shao-Yan Xi, Fang Wang, Hua-Dong Liao, Yuan-Zhong Yang, Wan-Ming Hu

**Affiliations:** ^1^Department of Pathology, Sun Yat-Sen University Cancer Center; State Key Laboratory of Oncology in South China; Collaborative Innovation Center for Cancer Medicine, Guangzhou, Guangdong, China; ^2^Department of Molecular Diagnosis, Sun Yat-Sen University Cancer Center; State Key Laboratory of Oncology in South China; Collaborative Innovation Center for Cancer Medicine, Guangzhou, Guangdong, China

## Abstract

The latest WHO guideline of CNS tumor defined a RELA fusion-positive ependymoma type with extremely poor prognosis, and the expression of L1CAM was correlated well with the presence of RELA fusion. However, the L1CAM protein expression in large sample gliomas other than ependymoma, its relationship with the RELA gene and its prognostic significance remained unknown. We examined the expression of L1CAM in 565 glioma cases (WHO grade I-IV). The L1CAM IHC-positive cases were selected to test RELA fusion with FISH break-apart probes. L1CAM was positive in 109 cases (19.29%) of all 565 glioma cases, with 18.27% in low-grade gliomas and 19.84% in high-grade gliomas, respectively. Unlike ependymoma, L1CAM protein expression was not correlated with the C11orf95-RELA fusion gene in other gliomas, but it had correction with the patient age (older than 45-year-old, *p* = 0.006), ATRX mutation (*p* = 0.003) and Ki67 (*p* = 0.007). High expression of L1CAM was an independent prognostic factor in our cohort. Further analysis demonstrated that L1CAM strong positive expression was significantly associated with poor prognosis in gliomas, both in our cohort (*p* < 0.001) and TCGA (*p* < 0.009) dataset. Although uncorrelated with C11orf95-RELA fusion, L1CAM was a significant poor prognostic marker in glioma patients. More aggressive treatment should be taken for these patients and L1CAM might be a promising therapeutic target in glioma.

## 1. Introduction

Glioma is the most common malignant and highly aggressive brain tumor, possessing the characteristics of infiltrating growth and easy recurrence. Glioblastoma (GBM) is one of the most lethal and aggressive brain tumors with extremely poor prognosis and high rates of recurrence. No effective therapeutic method except surgery, radiotherapy, and temozolomide chemotherapy is a major challenge in the treatment of GBM. Optimal utilization of traditional and novel targeting therapy modalities requires to explore novel molecular markers on this disease.

L1CAM (Cell Adhesion Molecule L1/CD171), a 200 kDa glycoprotein, belongs to the immunoglobulin supergene family and significantly involves in nervous system development, such as neuronal differentiation and migration. In the past few years, a lot of studies discussed the function and expression of L1CAM in human malignancies of different patient samples. It was a predictive factor of poor prognosis with vulvar cancer, endometrial cancer, gastric cancer, etc. [1]. However, only few studies researched L1CAM in glioma, it found to act as a putative role in the histogenesis of glioma, which conferred chemoresistance and stimulated glioma cell motility and proliferation [2–4].

In this study, the expression of L1CAM protein and its correlation with overall survival were investigated in a large series of 565 glioma samples from our cancer center, in order to further understand the expression and prognosis value of L1CAM in gliomas and its correlation with RELA gene and other important parameters.

## 2. Materials and Methods

### 2.1. Patient Selection and Sample Collection

In our study, 565 pathologically proven glioma specimens were obtained from Sun Yat-sen Cancer Center between 1998 and 2016. All the samples were obtained the informed consent of the patients. The series consisted of 24 cases of WHO I (pilocytic astrocytoma), 176 cases of WHO II (astrocytoma and oligodendroglioma), 159 cased of WHO III (anaplastic astrocytoma and oligodendroglioma), and 209 cases of WHO IV (glioblastoma). The ratio of male to female was 1.35 : 1. The median patient age at the time of primary surgery was 41 years (range 2-78 years). Median follow-up was 29 months (range 0-188 months). Tissue microarray was constructed as the method described previously [5]. All the patients had follow-up information and subjects with incomplete clinical data; preoperative death was not included in the current study. Overall survival (OS), calculated as the period from diagnosis until the date of death, was used for prognostic analysis in the current study.

### 2.2. Immunohistochemistry (IHC)

Immunohistochemistry was performed as described earlier. IHC for detection of L1CAM (mouse monoclonal antibody, clone UJ127.11; Sigma Aldrich, St. Louis, MO, USA; 1 : 1500), IDH1-R132H (clone H09, 1 : 50; Dianova, Hamburg, Germany), ATRX (1 : 500; Sigma-Aldrich, St. Louis, MO, USA), P53 (1 : 100; Dako, Carpinteria, CA) and Ki67 (1 : 100; Dako, Carpinteria, CA) was performed on an automated BenchMark Ultra (Ventana Medical systems, Roche, SW).

Immunohistochemical evaluation was independently conducted by two pathologists blinded for patient characteristics and outcome, with discrepancies resolved by consensus under a microscope for multi-viewing.

The result of positive L1CAM staining was used the adjusted Allred scoring system to evaluate the results of L1CAM expression, and the total value was 0-12 by positive ratio × staining intensity. On account of normal brain tissues can weakly express L1CAM, diffuse and strong staining localized in tumor cell membrane and cytoplasm was defined as highly positive. Normal kidney tissue was used as a positive control and vascular endothelial cell was an internal negative control.

### 2.3. Fluorescence In Situ Hybridization (FISH)

FISH break-apart probes were derived from BAC clones (BACPAC Resources, Oakland, CA), rearrangement in the context of chromothripsis splits the dual-color signals in prob sets for RELA, and a C11orf95-RELA fusion supratentorial ependymoma was set as a positive control.

### 2.4. Human Protein Atlas Database (HPA)

The Human Protein Atlas (HPA) is an interactive open-access database containing mRNA and protein expression data based on the integration of publicly available data from TCGA and data generated within the framework of the HPA (http://www.proteinatlas.org/) [6]. The TCGA cohort in HPA consisted of 153 glioblastoma patients with detailed clinical information was downloaded and analyzed with SPSS.

### 2.5. Statistical Analysis

Associations between categorical variables were evaluated by the use of 2 × 2 contingency tables and the chi-square (*χ*2) test. The association between L1CAM expression and clinic-pathological characteristics was assessed using the Spearman correlation coefficient. The Kaplan-Meier survival curves were conducted to estimate overall survival. Survival differences according to L1CAM expression were analyzed by the log-rank test. The influence of variables on survival was assessed using univariate and/or multivariate Cox regression analyses. All statistical analyses were performed with SPSS (version 16.0, Chicago, USA), and significance was defined as *p* < 0.05.

## 3. Results

### 3.1. L1CAM Was Highly Positive in 19% Gliomas and Associated with Patient Age, ATRX Status, and Ki-67 Index

**Figure 1:**
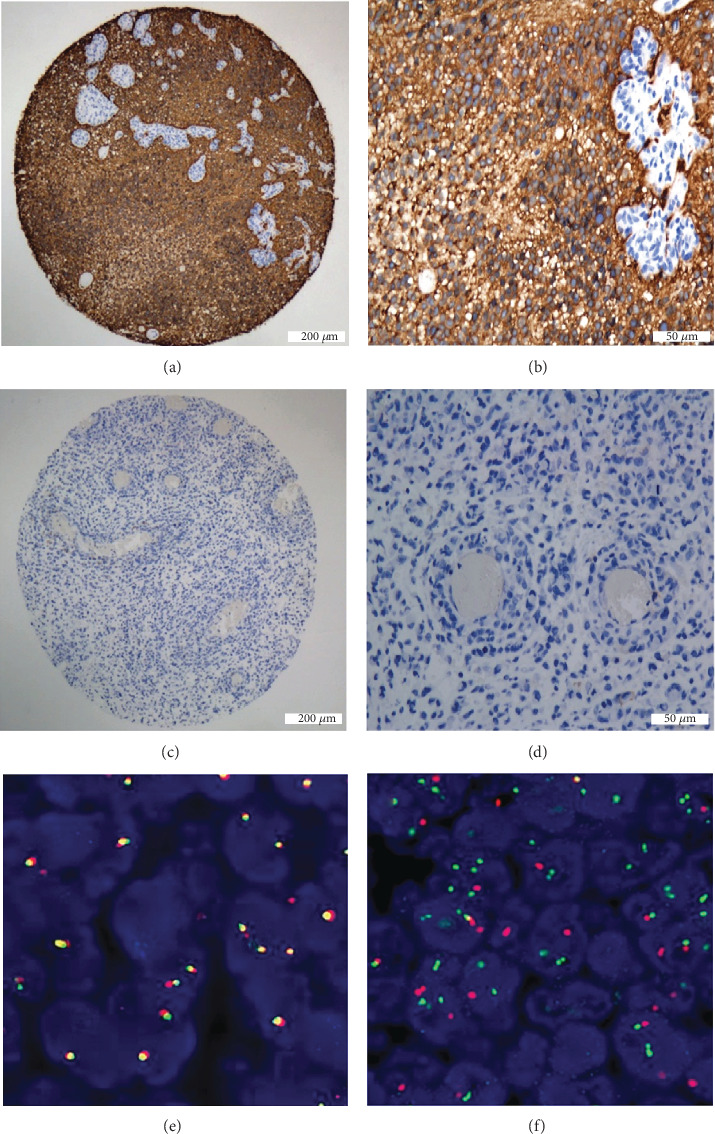
(a, b) L1CAM high expression case, strong cytoplasmic and membranous L1CAM immunoreactivity in a GBM case, and vascular endothelial cell was an internal negative control. 40x and 200x. (c, d) Negative case, with no staining, was found. 40x and 200x. (e) Overlapping probes (yellow) indicate an intact RELA gene, (f) but probe separation (red/green) occurs with rearrangement of the RELA gene (positive control).

The clinicopathological characteristics of the 565 glioma patient samples and the correlation with L1CAM expression were showed in Table 1. L1CAM was defined as highly positive cases when strong cytoplasmic and membranous staining expressed in tumor cells, and vascular endothelial cells were an internal negative control (Figures 1(a)–1(d)). Out of 565 glioma cases in our cohort, 109 (19%) tumors were found to be L1CAM highly positive, with 36 highly positive cases (18.27%) in low-grade glioma and 73 highly positive cases (19.84%) in high-grade gliomas, respectively. Specifically, all the L1CAM positive cases were diffuse gliomas, include 36 cases of WHO II (26 astrocytomas and 10 oligodendrogliomas), 28 cases of WHO III (18 anaplastic astrocytomas and 10 anaplastic oligodendrogliomas), and 45 cases of WHO IV (glioblastoma/GBM). Among all the 109 L1CAM positive gliomas, 44 cases were IDH mutated (10 astrocytomas, 10 oligodendrogliomas, 8 anaplastic astrocytomas, 10 anaplastic oligodendrogliomas, and 6 GBMs) and 65 cases were IDH wild-type (16 astrocytomas, 10 anaplastic astrocytomas, and 39 GBMs). High expression of L1CAM was correlated with patient age (*p* = 0.006), ATRX status (*p* = 0.003), and Ki-67 index (*p* = 0.007), but no correlation was found between L1CAM and gender, tumor location, WHO grade, IDH status, and P53 status (Table 1).

### 3.2. Different from Supratentorial Ependymoma, L1CAM Protein Expression Does Not Indicate RELA Gene Rearrangement in Other Gliomas

It is well known that high expression of L1CAM correlates well with the presence of RELA fusion in supratentorial ependymomas. In order to find its correlation with C11orf95-RELA fusion in other types of gliomas, we selected all the L1CAM IHC highly positive cases to do the FISH test using RELA break-apart probes. However, in 109 L1CAM positive cases (26 astrocytomas, 10 oligodendrogliomas, 18 anaplastic astrocytomas, 10 anaplastic oligodendrogliomas, and 45 glioblastomas), no one (0%) had a positive result of probe separation (red/green) (Figures 1(e) and 1(f)), indicating other mechanisms might lead to the strong expression of L1CAM in some glioma cases, rather than alteration of the RELA gene.

### 3.3. L1CAM Is an Independent Poor Prognostic Marker in Glioma

**Figure 2:**
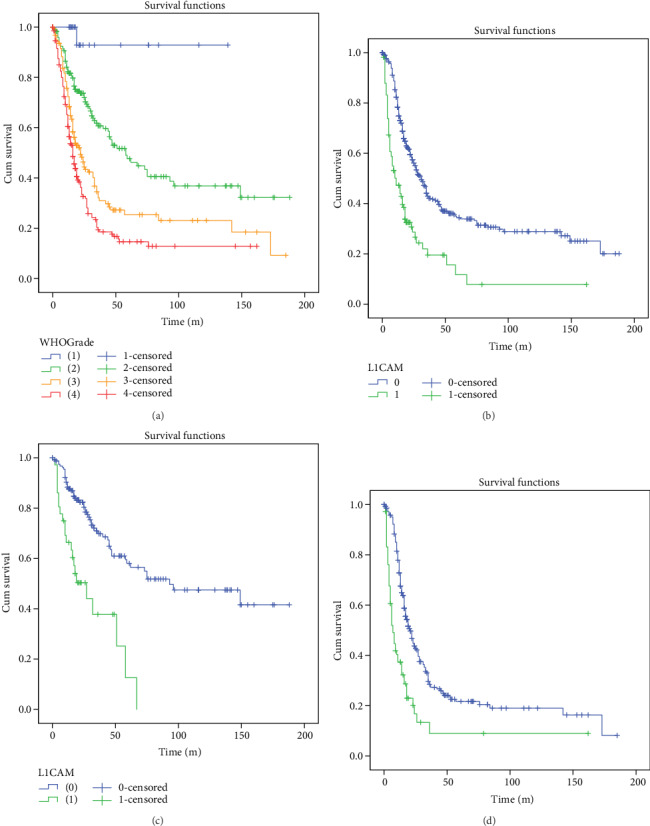
High L1CAM expression was associated with significantly shorter OS both in high-grade and low-grade gliomas (a) The OS of all glioma patients in our cohorts grouped by WHO grade. (b) The OS of L1CAM in all glioma patients. (c) The OS of L1CAM in low-grade glioma patients (WHO I and II). (d) The OS of L1CAM in high-grade glioma patients (WHO III and IV).

The prognostic value of L1CAM expression was analyzed using univariate and multivariate analysis. In univariate analysis, the results indicated that a notable correlation was discovered between the overall survival and these clinicopathological prognostic factors, including tumor location, age at diagnosis, tumor recurrence, and clinical stage (*p* < 0.05, Table 2). Among them, older age (>45), high WHO grade (III/IV), IDH wildtype, high P53 expression, high Ki67 index, and L1CAM strong expression were risk factors for unfavorable OS. Multivariate analysis showed that L1CAM was independently associated with shorter OS (HR: 1.528, 95% CI: 1.984-3.412, *p* < 0.001) after adjustment for other risk factors. In addition, more than 45 years old at diagnosis, WHO high grade, and IDH wildtype were also served as independent prognostic factors for overall survival (Table 2) in multivariate COX analysis. In Kaplan-Meier analysis, survival curves showed that the L1CAM highly positive group had the poor OS in all gliomas (WHO I-IV), for the mean survival time of the L1CAM negative group (70.4 months, 95% CI: 61.7-79.2) was significantly longer than the mean survival time of L1CAM positive patients (28.3 months, 95% CI: 16.2-40.4). In stratification analysis, L1CAM was also a significant poor prognostic marker both in low-grade glioma (WHO I and II) and high-grade glioma (WHO III and IV) (Figure 2).

### 3.4. Validation of L1CAM Expression in HPA and Its Prognostic Significance in TCGA

**Figure 3:**
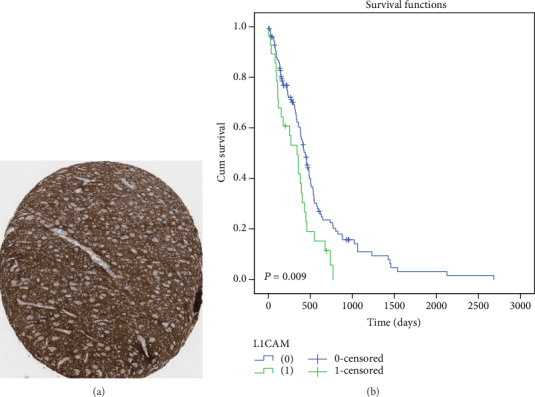
(a) L1CAM strong expression IHC images in HPA. Image was obtained from: https://www.proteinatlas.org/ENSG00000198910-L1CAM/pathology/tissue/glioma#img. (b) Prognostic values of L1CAM' mRNA expression in 153 glioblastoma patients in TCGA, for L1CAM high expression patients (FPKM cutoff value = 5.9) with a poor prognosis (*p* = 0.009).

To further confirm our results, we queried the L1CAM expression in Human Protein Atlas (HPA). In the HPA database, the protein expression score is a combination of staining intensity and stained cell proportion and is divided into four levels: negative, low, medium, and high. Intriguingly, we also detected the strong expression of L1CAM protein in some glioma tissues in HPA (Figure 3(a)), with 2 of 12 (16.67%) glioma patients show high expression of L1CAM. Based on survival data from TCGA, we compared the overall survival between patients with high L1CAM expression and low L1CAM expression. As the same as our results, TCGA data also showed the high expression group had remarkably shorter OS (Figure 3(b)).

## 4. Discussion

It is generally known that malignant glioma is a common and devastating disease associated with poor median survival time and no effective targeted cure strategies. It makes sense to further understand the genetic factors of this aggressive disease and explore novel targeted treatments. It is reported that L1CAM is related to the progression of several kinds of solid cancer, including ovarian cancer [7], colon cancer [8], gastric cancer [9], malignant melanoma [10], breast cancer [11], and pancreatic cancer [12]. As the characteristics of extracellular and intracellular domains, L1CAM has been seemingly involved in tumor proliferation, migration, and invasion.

Indeed, L1CAM was first discovered as a novel neuronal cell surface component involved in cell adhesion by Maness and Schachner in 1984 [13, 14]. There are quite a lot of researches showing that L1CAM has been involved in a plenty of neural events [15], such as neuron adhesion, cerebellar granule cell migration [16], outgrowth of neurites and fasciculation [17, 18], myelination [19], and synaptic plasticity [20]. However, only a few studies focused on glioma. Anderson [21] revealed that L1CAM acted through integrin, focal adhesion kinase (FAK), and fibroblast growth factor receptor (FGFR) signaling pathways in glioblastoma derived cell lines to increase their motility, proliferation, and invasiveness. In another study, Held-Feindt [3] showed that TGF-*β*1 signaling regulated L1CAM expression in glioblastoma, and L1CAM conferred resistance to temozolomide. It seemed L1CAM acted different molecular signals in glioma compared with other solid cancers. Takeshi [22] reported that L1CAM triggered the ERK signaling pathway to regulate gastric cancer cell proliferation, another study showed that L1CAM was potentially involved in epithelial-mesenchymal transition and modulated MAPK/AKT signaling pathways in nonsmall cell lung cancer [23], and a study from Germany [24] summarized L1CAM might signal via two additional mechanisms: “forward” signaling via regulated intramembrane proteolysis and “reverse” signaling via the activation of the transcription factor nuclear factor (NF)-*κ*B, which was demonstrated by Pietsch and Parker in ependymoma [25, 26]. It was worth noting that expression of L1CAM correlated well with the presence of a RELA fusion in supratentorial ependymomas, and this new type presented in 2016 WHO blue book of CNS tumors had the worst outcome in ependymomas. However, we did not find C11orf95-RELA fusion in our L1CAM positive glioma cases, indicating the identification of the exact signaling pathway in glioma remained critical goals and needed further studies. Intriguingly, L1CAM was also found in glioma stem cells; Bao [27] showed that L1CAM was overexpressed in CD133+ glioblastoma cells. Cheng and colleagues [28] provided supporting evidence for this phenomenon, showing that L1CAM was highly expressed in a population of glioblastoma stem cells in the invasive fronts of primary GBMs, and targeted L1CAM might reduce GBM cancer invasion and tumor recurrence. Regarding molecules associated with L1CAM, Yang [29] demonstrated the released L1CAM ectodomain, likely by ADAM10 proteolysis, that stimulated the cell migration possibly through binding to cell surface receptors to activate the FAK signaling pathway. Mohanan [4] further confirmed L1CAM stimulated high-grade glioma cell motility and proliferation through the fibroblast growth factor receptor (FGFR). Anderson [21] suggested that inhibitors of FGFR have the potential to decrease the aggressiveness of high-grade gliomas expressing L1CAM. In addition, the previous researches had also shown cytokine neuregulin 1 (Nrg1) could upregulate the L1CAM expression to enhancing the migration of glioma cells [30], and TGF-*β*1 could mediate L1CAM expression, then led to the downregulation of caspase-8 and apoptosis resistance [3]. In addition, we identified 134 core molecules that directly or indirectly interacted with L1CAM in three databases (Supplementary Materials). Expression of L1CAM correlated well with the presence of a RELA fusion in supratentorial ependymomas. However, no literature researched the relationship of L1CAM and common molecular markers for diffuse gliomas (IDH1, IDH2, 1p/19q, ATRX, P53, TERT, BRAF, H3F3A), except we found ATRX status was correlated with L1CAM but no correlation between L1CAM and IDH/P53 status. It needs to be further explored with more researches in the future.

Hitherto, a lot of studies were published on L1CAM prognostic value in a variety of tumors in larger patient groups. For instance, Mina Fogel [31] and his workmate also revealed that the overexpression of L1CAM was associated with poor prognosis in ovarian and uterine carcinomas and could sever as a new factor for predicting patient survival and disease progresses. Dellinger [32] found L1CAM expression was an independent predictor of poor survival in endometrial cancer and was associated with advanced stage, high-risk endometrial cancer. Chen [9] indicated L1CAM overexpressed in gastric cancer and associated with poor prognosis and played an important role in the progression and metastasis of gastric cancer. Tischler [23] found a subset of nonsmall cell lung cancer with vessel tropism and increased metastasis aberrantly expresses L1CAM, and it served as a novel poor prognostic marker. In general, L1CAM expression was associated with poor prognosis, tumor progression, and metastasis to lymph nodes in nearly all solid tumors. In addition, the Human Protein Atlas database also indicated that L1CAM was a prognostic marker in endometrial cancer (unfavorable), lung cancer (unfavorable), renal cancer (unfavorable), head and neck cancer (unfavorable), and other solid cancers. However, limited articles researched the prognosis significance of L1CAM in glioma, except ependymoma as discussed above. In our study, L1CAM was found to be a significant poor prognosis in glioma, and it was an independent prognostic factor in multivariate Cox analysis, indicating its prognostic importance in glioma. Additional research is required to confirm our findings.

Due to its importance for tumor progression and promotes cell motility, invasion, and metastatic formation, L1CAM might be a promising new target molecule for antibody-based therapy of cancers, and therapy experiments in xenotransplanted mice were successfully performed in ovarian, pancreatic, or cholangiocarcinoma tumors targeted L1CAM [33–35]. It was suggested that L1CAM might also be a promising individualized therapeutic target in glioma. Moreover, it might be used in the monitoring of tumor progression and therapeutic efficacy. A recent study [36] evaluated the presence of L1CAM in cyst fluid from glioblastoma and demonstrated high levels of L1CAM in the cyst fluid of glioblastoma, for the mean levels of L1CAM in tumor cyst fluid were significantly higher in glioblastoma than in CSF of control patients, indicating soluble L1CAM might represent a motility promoting molecule in glioma progression.

In summary, L1CAM was found to be a significant marker in predicting the prognosis of glioma patients, but unlike ependymoma, it was not correlated with RELA in other gliomas. In addition, L1CAM may be a promising therapeutic target and monitoring index in glioma patients.

## Figures and Tables

**Table 1:** Clinicopathological characteristics in relation to L1CAM expression.

	Total	L1CAM		L1CAM		*P*
		Negative		Positive		
Total	565	456	(80.71%)	109	(19.29%)	
*Gender*						0.400
Male	323	259	(80.19%)	64	(19.81%)	
Female	242	197	(81.40%)	45	(18.60%)	
*Age*						*0.006*
<=45	343	289	(84.26%)	54	(15.74%)	
>45	222	167	(75.23%)	55	(24.77%)	
*Location*						0.583
Supratentorial	517	413	(79.88%)	104	(20.12%)	
Subtentorial	48	43	(89.58%)	5	(10.42%)	
*WHO grade*						0.371
Low-grade (I and II)	197	161	(81.73%)	36	(18.27%)	
High-grade (III and IV)	368	295	(80.16%)	73	(19.84%)	
*IDH*						0.260
Mutated	240	196	(81.67%)	44	(18.33%)	
Wildtype	325	260	(80.00%)	65	(20.00%)	
*ATRX*						*0.003*
Mutated	239	215	(89.96%)	24	(10.04%)	
Wildtype	326	241	(73.93%)	85	(26.07%)	
*P53*						0.181
<=10%	245	193	(78.78%)	52	(21.22%)	
>10%	320	263	(82.19%)	57	(17.81%)	
*Ki67*						*0.007*
<=10%	194	168	(86.60%)	26	(13.40%)	
>10%	371	288	(77.63%)	83	(22.37%)	

**Table 2:** Univariate and multivariate analysis for overall survival in glioma.

Variable	*Univariate analysis*		*Multivariate analysis*	
	Hazard ratio (95% CI)	*P* value	Hazard ratio (95% CI)	*P* value
Gender (male)	1.176 (0.941~1.470)	0.155		
Age (years >45)	1.808 (1.449~2.256)	*<0.001*	1.182 (0.926-1.509)	0.178
Location (Supratentorial)	1.172 (0.921~1.493)	0.197		
WHO grade (high)	2.582 (1.993~3.343)	*<0.001*	*1.662 (1.182-2.337)*	*0.003*
IDH (mutated)	0.336 (0.262~0.432)	*<0.001*	*0.427 (0.327-0.557)*	*<0.001*
ATRX (mutated)	1.104 (0.881~1.383)	0.392		
P53 (>10%)	1.315 (1.047~1.652)	*0.018*	1.216 (0.949-1.560)	0.123
Ki67 (>10%)	2.464 (1.907~3.182)	*<0.001*	1.269 (0.901-1.786)	0.173
L1CAM (positive)	2.503 (1.932~3.241)	*<0.001*	*1.528 (1.984-3.412)*	*<0.001*

## Data Availability

All the data are in the article. No additional data available.

## Supplementary Materials

Supplementary Materials Supplementary Figure: molecules associated with L1CAM in TCGA, Gravendeel and Rembrandt databases. (A) We first screened out 359 genes co-related to L1CAM in the TCGA, Rembrandt, and Gravendeel database(r>0.5). The protein-protein interaction (PPI) network was analyzed established by STRING database (http://string-db.org/) with a criterion of combined score >0.4 considered to be a significant result. We finally identified 134 core genes that directly or indirectly interact with L1CAM, and used software Cytoscape 3.7.2 to reveal the PPI network visualization of protein interactions. (B) ANK1, ANK3, CA10, NCAM2, SH3GL2, SPTBN2 and STMN2 are 7 direct interaction molecules associated with L1CAM.
