# Supplementary figures and images for: L1CAM High Expression Associates with Poor Prognosis in Glioma but Does Not Correlate with C11orf95-RELA Fusion
Source: Biomed Res Int. 2020 May 16;2020:1353284. doi: 10.1155/2020/1353284 (PMC7251433; doi:10.1155/2020/1353284)

**L1CAM-Remdrandt**

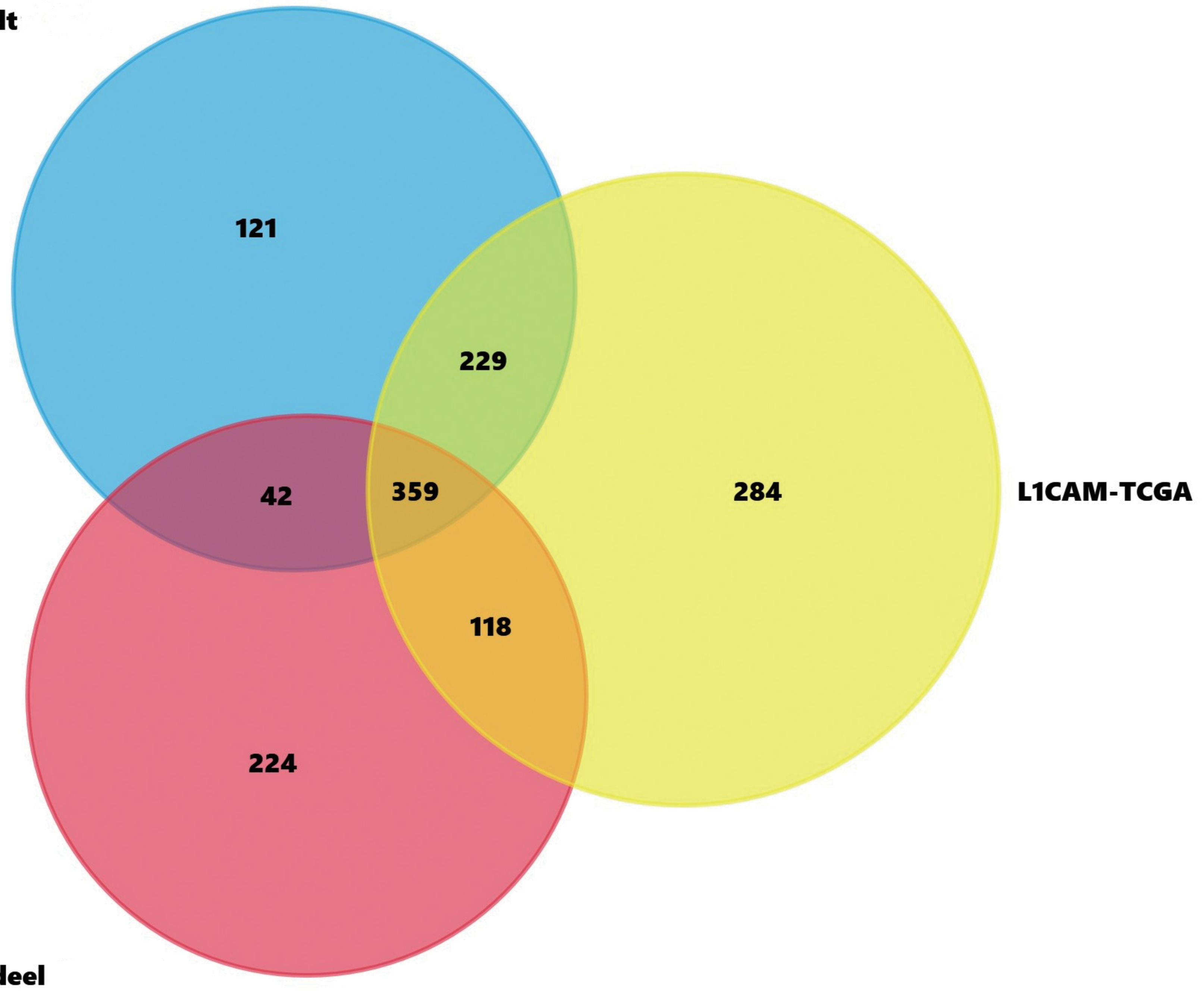

**L1CAM-Gravendeel**

**L1CAM-TCGA**

Supplement: Supplementary Materials — Supplementary Figure: molecules associated with L1CAM in TCGA, Gravendeel and Rembrandt databases. (A) We first screened out 359 genes co-related to L1CAM in the TCGA, Rembrandt, and Gravendeel database(r>0.5). The protein-protein interaction (PPI) network was analyzed established by STRING database (http://string-db.org/) with a criterion of combined score >0.4 considered to be a significant result. We finally identified 134 core genes that directly or indirectly interact with L1CAM, and used software Cytoscape 3.7.2 to reveal the PPI network visualization of protein interactions. (B) ANK1, ANK3, CA10, NCAM2, SH3GL2, SPTBN2 and STMN2 are 7 direct interaction molecules associated with L1CAM. [file 1353284.f1.zip › 1353284.f1/Figure SA_BMRI_3072517.pdf]

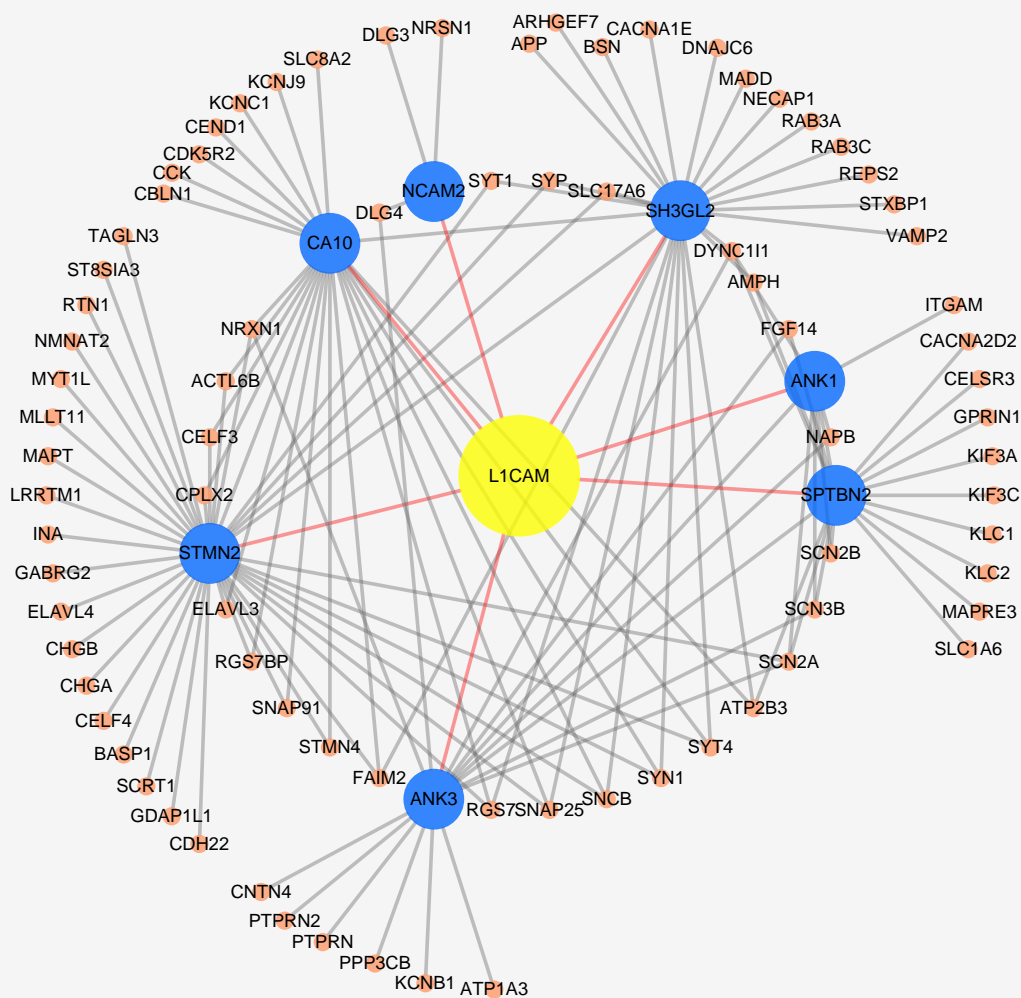

Supplement: Supplementary Materials — Supplementary Figure: molecules associated with L1CAM in TCGA, Gravendeel and Rembrandt databases. (A) We first screened out 359 genes co-related to L1CAM in the TCGA, Rembrandt, and Gravendeel database(r>0.5). The protein-protein interaction (PPI) network was analyzed established by STRING database (http://string-db.org/) with a criterion of combined score >0.4 considered to be a significant result. We finally identified 134 core genes that directly or indirectly interact with L1CAM, and used software Cytoscape 3.7.2 to reveal the PPI network visualization of protein interactions. (B) ANK1, ANK3, CA10, NCAM2, SH3GL2, SPTBN2 and STMN2 are 7 direct interaction molecules associated with L1CAM. [file 1353284.f1.zip › 1353284.f1/Figure SB_BMRI_3072518.pdf]
